# Isolation and characterization of mesenchymal stem cells from human fetus heart

**DOI:** 10.1371/journal.pone.0192244

**Published:** 2018-02-08

**Authors:** Venkata Naga Srikanth Garikipati, Saurabh Pratap Singh, Yamuna Mohanram, Ashwani Kumar Gupta, Deepa Kapoor, Soniya Nityanand

**Affiliations:** 1 Stem Cell Research Facility, Department of Hematology, Sanjay Gandhi Post-Graduate Institute of Medical Sciences, Lucknow, India; 2 General Hospital, Sanjay Gandhi Post-Graduate Institute of Medical Sciences, Lucknow, India; University of Kansas Medical Center, UNITED STATES

## Abstract

**Background:**

Mesenchymal stem cells (MSCs) are promising cells for cardiovascular regenerative medicine. However, their potential may be limited, because of their restricted cardiovascular differentiation potential and decline in their number and functional characteristics with increasing donor age. We have previously shown that rat fetus heart harbors primitive MSCs and administration of these cells improved left ventricular (LV) function after ischemia/reperfusion injury in rats. To evaluate their potential as a new cell type for clinical cardiovascular cell therapy, we have undertaken this study on the isolation and characterization of human fetal cardiac MSCs (hfC-MSCs).

**Methods:**

MSCs were isolated from the heart of five 14-16-week-old aborted human fetuses and studied for their growth characteristics, karyotypic stability and senescence over successive passages, expression of mesenchymal and embryonal markers by flow cytometry and immunocytochemistry, constitutive expression of cardiovascular genes by RT-PCR, differentiation into cells of the cardiovascular lineage and their immunomodulatory properties.

**Results:**

The hfC-MSCs grew as adherent monolayer with spindle shaped morphology and exhibited rapid proliferation with an average population doubling time of 34 hours and expansion to up to more than 80 population doublings with maintenance of a normal karyotype and without senescence. Immunophenotyping showed that they had similar phenotype as human bone marrow mesenchymal stem cells (hBM-MSCs) expressing CD73, CD90, CD105 and lacking expression of CD31, CD34, CD45, HLA-DR. However, hfC-MSCs expressed significantly higher levels of CD117 and SSEA-4 compared to hBM-MSCs. In addition, hfC-MSCs expressed the embryonal markers Oct-4, Nanog and Sox-2 as compared to hBM-MSCs. Further, hfC-MSCs had significantly higher expression of the cardiovascular genes viz. ISL-1, flk-1, GATA-4, NKX2.5 and MDR-1 as compared to hBM-MSCs, and could be differentiated into major cardiovascular cells (cardiomyocytes, endothelial cells, smooth muscle cells). Interestingly, hfC-MSCs markedly reduced T-lymphocyte proliferation with an increased secretion of TGF-β and IL-10.

**Conclusions:**

Our results show that human fetus heart is a novel source of primitive MSCs with cardiovascular commitment which may have a potential therapeutic application in cardiovascular regenerative medicine.

## Background

Stem cell therapy has shown immense potential for cardiac repair and regeneration. In this context, adult bone marrow derived mesenchymal stem cells (BM-MSCs) represent the most studied cell type for cardiac repair due to their unique features including the ease in their isolation/expansion, their paracrine activity to induce neovascularization post ischemic injury and most importantly due to their immunomodulatory properties [1–5]. However, clinical trials on MSC therapy for cardiac disease have resulted in modest benefits [6].

A recent study has shown that adult cardiac mesenchymal stromal cells compared to hBM-MSCs constitutively express cardiovascular genes and differentiate into cardiovascular cells both *in vitro* and *in vivo*, suggesting the superiority of cardiac specific cells for cardiac repair and regeneration [7]. However, it is now increasingly evident that chronological age affects regenerative ability of stem cells to repair the heart after injury [8–10]. A recent study has shown that neonatal cardiac stem cell therapy outperforms adult cardiac stem cell therapy in functional improvement after myocardial infarction (MI) in rodents [11]. Furthermore, we have recently shown that early developmental stage rat heart to be a superior source of primitive stem cells expressing typical mesenchymal stem cell, embryonal and cardiovascular markers. We labelled these cells as rat fetal cardiac mesenchymal stem cells (rfC-MSCs) and upon induction the rfC-MSCs could differentiate into major cardiovascular cell types including cardiomyocytes, endothelial cells and smooth muscle cells. When transplanted in rat model of ischemia/reperfusion myocardial injury, rfC-MSCs reduced apoptosis, fibrosis and improved cardiac function [12, 13]. We thus hypothesized that MSCs derived from human fetus heart may also possess such primitive characteristics and be a suitable/superior cell source for cardiac regeneration. Therefore, the aim of the present study was the isolation and characterization of human fetal cardiac MSCs (hfC-MSCs).

## Materials and methods

### Isolation and culture of resident human fetal cardiac mesenchymal stem cells (hfC-MSCs)

Fetal tissues (n = 5) were obtained from women undergoing elective termination of second-trimester (14–16 weeks) pregnancies in Sanjay Gandhi Post Graduate Institute of Medical Sciences, under conditions where it was clear that tissues would otherwise be discarded. Pregnant women gave written informed consent for the use of tissues for research purposes. Fetal gestational age was determined by crown–rump length measurement on ultrasound. All tissues were collected after obtaining consent from mothers for their use for research purposes and protocol was approved by the Sanjay Gandhi Post Graduate Institute of Medical Sciences, Institutional Ethics Committee.

The hearts removed aseptically from second trimester fetuses were minced, and digested with 1 mg/ml collagenase type-IV (Worthington Biochemical) in serum free α-MEM medium for 30 min at 37°C with intermittent stirring. After two washes with α-MEM, the digested tissue was cultured at 37°C in a 5% CO_2_ in 25 cm^2^ tissue culture flasks (Becton, Dickinson) in complete culture medium consisting of α-MEM medium, 2mg/ml of Glutamax (Gibco-Invitrogen), 16.5% fetal bovine serum (Hyclone) and bacteriostatic level of penicillin-streptomycin (Gibco-Invitrogen). After 48 hours, the culture medium was changed and non-adherent cells were removed. Within 72 hours of change of medium a sub-confluent growth of adherent cells was obtained. These adherent cells were harvested by trypsinization (0.05% Trypsin-EDTA (Gibco -Invitrogen) and the cells were expanded in larger flasks up to 15 passages.

Three independent hfC-MSC cultures were set up from cells and studies given below were carried out at passages 3–5 passages.

### Isolation and culture of human bone marrow mesenchymal stem cells (hBM-MSCs)

Bone marrow aspirates were obtained from the posterior superior iliac crest of nutritional anemic donors registered in Sanjay Gandhi Postgraduate Institute of Medical Sciences, after obtaining informed consent and protocol was approved by the Sanjay Gandhi Post Graduate Institute of Medical Sciences, Institutional Ethics Committee. The samples were subjected to density gradient centrifugation using Ficoll-Paque for the isolation of the mononuclear cell population. The enriched mononuclear cell population was then cultured at 37°C in a 5% CO_2_ in 25 cm^2^ tissue culture flasks (Becton Dickinson) in complete culture medium consisting of α-MEM medium, 2mg/ml of Glutamax (Gibco-Invitrogen), 16.5% fetal bovine serum (Hyclone) and bacteriostatic level of penicillin-streptomycin (Gibco-Invitrogen). Following 48 hours of culture, the non-adherent cells were removed and the cells were replenished with fresh cell culture media. Cell attachment and spreading were observed within 72 hours of change of culture. Human BM-MSC cultures were set up from the cells and studies here below were carried out between passages 3–5.

### Growth kinetics

The growth kinetics of hfC-MSCs and hBM-MSCs were evaluated by plating the cells in triplicates at a concentration of 1000 cells per cm^2^ in 10-cm^2^ petri-dishes, harvesting them daily up to day 7 and plotting a growth curve between number of live cells obtained and the time. The number of population doublings of hfC-MSCs was determined by counting the number of adherent cells at the start and end of each passage. The population doubling time was calculated following the standard formula (log_*N*_/log_2_)/*t*, where *N* is the number of cells at confluence divided by the initial number of cells, and *t* is the number of hours in culture.

### Karyotyping

hfC-MSCs were grown in 25 cm^2^ tissue culture flasks for 3 days and treated with 10 μg/μl of colcemid (Sigma-Aldrich) for 30 min followed by hypotonic shock (60mM KCl) and finally fixed with methanol/acetic acid (3:1). Chromosomes of at least 10 metaphases were counted under microscope. A karyotypic analysis was performed at 15^th^ passage.

### Flow cytometry

hBM-MSCs or hfC-MSCs were stained with following anti-human monoclonal antibodies labelled with Fluorescence isothyocyanate (FITC) or phycoerythrin (PE): CD73-PE, CD90-PE, CD105-PE, CD117-PE, SSEA-4-PE, CD31-FITC, CD34-FITC, CD45-FITC, HLA-DR-FITC (all from Serotec), or isotype matched control monoclonal antibodies (Becton Dickinson). Stained cells were analyzed in Flow Cytometer (FACS Calibur, Becton Dickinson).

### Real time PCR

Expression of ISL-1, FLK-1, GATA-4, NKX2.5 and MDR-1 in hfC-MSCs and in hBM-MSCs was determined by real time PCR. RNA of the cells was extracted using RNeasy Mini RNA isolation kit (Gibco-Invitrogen) 1μg of total RNA was reverse transcribed into cDNA using random hexamers (Gibco-Invitrogen). The gene expression was analyzed for the following genes using primers from (MWG Biotech) **(Table 1)**. The resulting cDNA was quantified by real-time PCR using SYBR Green PCR Master Mix (Roche) and using Roche Light Cycler 4800 (Roche). GAPDH was used as the house keeping gene for normalization. The Primer pair sequence listed in Table 1 was used.

**Table 1:** Primers used in this study.

S.No	Gene	Primers(5’–3’)
1	Gata-4	(f)5’-GCTCCTTCAGGCAGTGAGAG-3’
		(r)5’-CTGTGCCCGTAGTGAGATGA-3’
2	Nkx2.5	(f)5’-GACCCTCGGGCGGATAAGAAAGA-3’
		(r)5’-CGGGATAGGGGTAGGCGTTGTAG-3’
3	Isl-1	(f)5′-AAACTAATATCCAGGGGATGACAGG-3′
		(r)5′-CTCAGTACTTTCCAGGGCGG-3′
4	Flk-1	(f)5’-GTACCAAACCATGCTGGATTGC3’
		(r)5’-CTTGCAGGAGATTTCCCAAGTG3’
5	MDR-1	(f)5’-CCTCTCTCTTGCTCTCAGTAT-3’
		(r)5’-GTATCCGTTGTGGATCTGACA-3’
6	β-actin	(f)5'-GCTCGTCGTCGACAACGGCTC-3’
		(r)5'-CAAACATGATCTGGGTCATCTTCTC-3'

### Immunocytochemistry

Expression of Oct-4, NANOG and SOX-2 by hfC-MSCs and hBM-MSCs were studied by immunocytochemistry. The cells were fixed with 4% para-formaldehyde (Sigma Aldrich) in phosphate buffered saline (PBS), pH 7.4, for 1 hr at room temperature. The fixed cells were incubated overnight at 4°C with following primary antibodies: Oct-4, NANOG, and SOX-2 (ES Cell characterization kit; Chemicon), diluted 1:50. After washing with PBS, cells were incubated with 1:500 diluted IgG (Fab) _2_ FITC as secondary antibody (Abcam) and stained with Hoechst dye. The pictures were taken using fluorescent microscope (Nikon 80i, Japan).

### Adipogenic differentiation

hfC-MSCs were treated with adipogenic medium consisting of DMEM medium (Gibco-Invitrogen) containing 10% FBS (Hyclone), 500mM Isobutylmethylxanthine, 1mM dexamethasone, 10mg/ml insulin and 100mM Indomethacin (Adipogenesis kit, Chemicon). The control cells were treated with DMEM medium (Gibco-Invitrogen) containing 10% FBS (Hyclone) alone. After 18 days the experimental and control cells were fixed and stained with Oil-Red O Stain to visualize fat droplets in the cells.

### Osteogenic differentiation

hfC-MSCs were treated with osteogenic medium consisting of DMEM medium (Gibco- Invitrogen) containing 10% FBS (Hyclone), 1 mM dexamethasone, 10 mg/ml glyceraldehyde 3-phosphate, and 0.1mM Ascorbic acid (Osteogenesis kit, Chemicon). The control cells were treated with DMEM medium (Gibco-Invitrogen) containing 10% FBS (Hyclone) alone. After 21 days, the experimental and control cells were fixed with 4% para-formaldehyde and stained with Alizarin-Red Stain to visualize mineralization.

### Cardiomyogenic differentiation

hfC-MSCs were treated with cardiogenic medium consisting of complete culture medium containing 10μM 5-azacytidine (Sigma-Aldrich). hfC-MSCs were treated with complete medium alone. After incubating for 24h, the experimental and control cells were washed twice with PBS and further incubated in complete culture medium. The medium was changed every three days and the experiment was terminated four weeks after 5-azacytidine treatment. The cardiomyogenic nature of the cells was characterized by immunocytochemistry using primary antibodies against cardiac troponin T (cTnT) (1:500 dilution) (Serotec). After washing with PBS, cells were incubated with 1:500 diluted IgG (Fab) _2_ FITC as secondary (Abcam) and stained with Hoechst dye (Sigma). The pictures were taken using fluorescent microscope (Nikon 80i, Japan).

### Endothelial differentiation

hfC-MSCs were seeded onto glass cover slips in 6 well plate (Becton Dickinson). The cells on cover slips at 70% confluence were treated with endothelial differentiation medium or complete culture medium (control cells). The endothelial differentiation medium consisted of complete culture medium supplemented with 100ng/ml vascular endothelial growth factor (R&D systems) and 1X mercaptoethanol (2-ME) (Gibco-Invitrogen). The differentiation medium was changed every alternate day for three weeks. The endothelial cells differentiation was characterized by immunocytochemistry using 1:500 diluted primary antibody against CD31 (Serotec). After washing with PBS, cells were incubated with 1:500 diluted IgG (Fab) _2_ FITC as secondary (Abcam) and stained with Hoechst dye (Sigma). The pictures were taken using fluorescent microscope (Nikon).

### Smooth muscle cell differentiation

hfC-MSCs were seeded onto glass cover slips in 6 well plate (Becton Dickinson). The cells on cover slips at 70% confluence were treated with smooth muscle differentiation medium or complete culture medium (hfC-MSCs). The smooth muscle differentiation medium consisted of complete culture medium supplemented with 50ng/ml TGF-β (R&D systems). The differentiation medium was changed every alternate day for three weeks. The smooth muscle differentiation was characterized by immunocytochemistry using 1:100 diluted primary antibody against smooth muscle myosin heavy chain (SM-MHC) (Serotec). After washing with PBS, cells were incubated with 1:500 diluted IgG (Fab) _2_ FITC as secondary (Abcam)) and stained with Hoechst dye (Sigma). The pictures were taken using fluorescent microscope (Nikon).

### Peripheral blood mononuclear cell isolation

Peripheral blood mononuclear cells (PBMCs) were obtained from 5 healthy volunteers upon informed consent and protocol was approved by the Sanjay Gandhi Post Graduate Institute of Medical Sciences, Institutional Ethics Committee. Briefly, peripheral blood sample was diluted with phosphate buffered saline in an equal ratio and then layered onto Histopaque (specific gravity 1.077; Sigma-Aldrich) and centrifuged at 2100 rpm for 30 min without brake. PBMCs were collected from the buffy coat and washed twice with PBS. Cell viability was checked with trypan blue dye.

### Cell proliferation assay

hfC-MSCs were seeded at a density of 5×10^3^ to 5×10^4^ cells per well in 96-well plate and cultured in high glucose alpha MEM, 10% FBS, 100 U/ml penicillin/streptomycin. After 24 h, 10μg/ml mitomycin C (MMC; Sigma–Aldrich) was added to inhibit MSC proliferation, and cells were incubated for 2 h at 37°C. Peripheral blood mononuclear cells (PBMCs) were isolated as described above. 1 x10^5^ PBMCs per well were added and stimulated with 5μg/ml phytohemagglutinin (PHA; Sigma–Aldrich). PHA-activated PBMCs were cultured in the presence or absence of irradiated MSCs. Cultures were incubated for 96 hours at 37°C in a humidified atmosphere of 5% CO_2_. Supernatant (100μl) was removed from each triplicate well and pooled for cytokine analysis. Cultures were pulsed with 1 Ci/well [3H] thymidine diluted in RPMI 1640 medium, incubated for another 18 hours at 37°C in a humidified atmosphere of 5% CO_2_, and then harvested onto glass fiber filters. Thymidine incorporation was measured using a liquid scintillation counter (Wallac). Results were expressed as the mean counts per minute for each triplicate culture.

### ELISA

To determine the levels of TGF-β and IL-10 in the cell-free culture supernatant, commercial ELISA kits (R&D systems) were used per the manufacturer’s instructions.

### Statistical analysis

Results were calculated for our data and presented as mean ± SE where indicated. Statistical significance was defined as *p* < 0.05 using Student’s *t*-test or analysis of variance (ANOVA) Software.

## Results

### Morphology, growth kinetics and karyotype of hfC-MSCs

**Fig 1:**
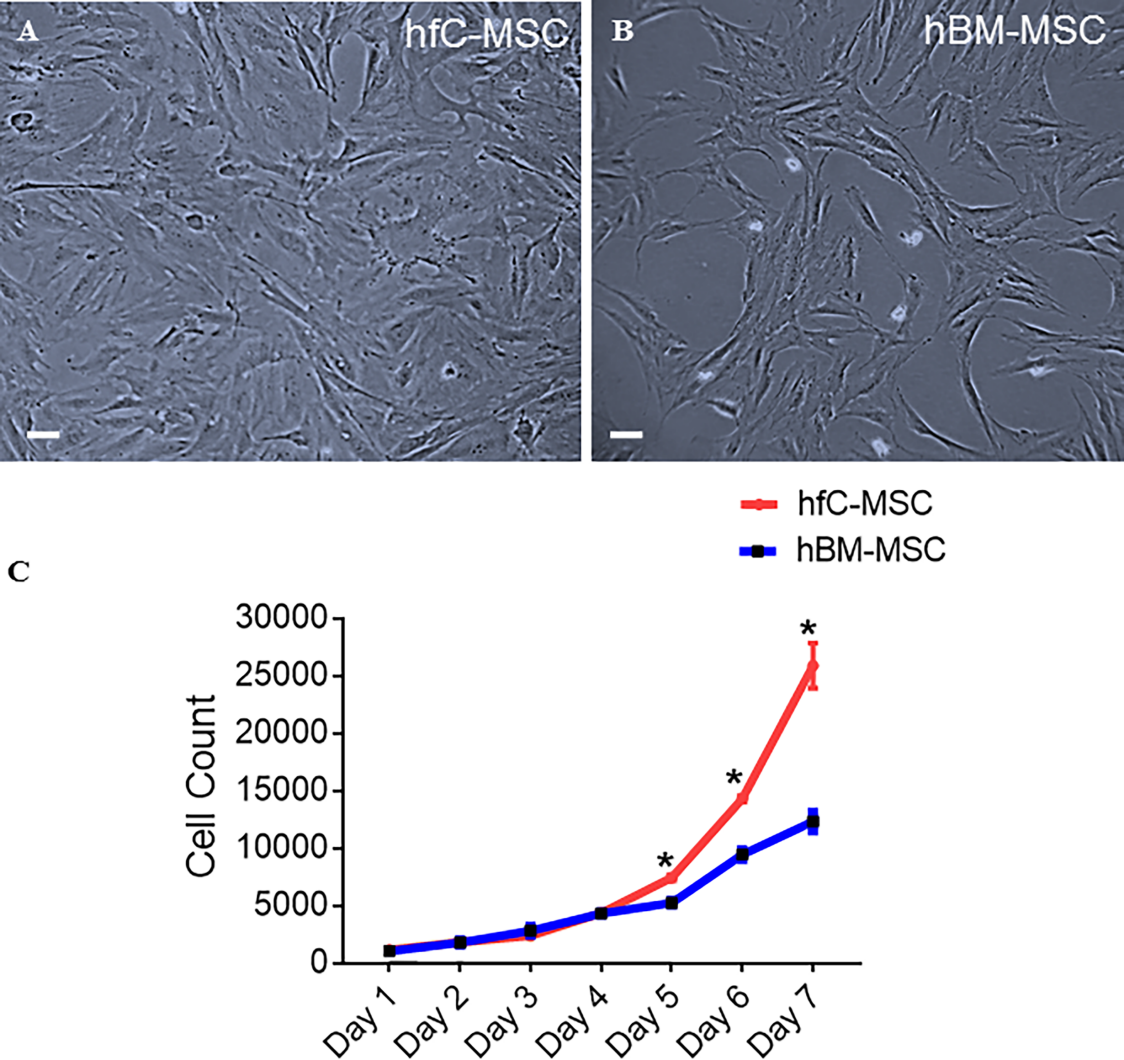
Morphology and Growth Kinetics of hfC-MSCs compared to hBM-MSCs. Representative photomicrographs (10X, 20μm) of (A) Human fetal cardiac mesenchymal stem cells (hfC-MSCs); (B) Bone marrow mesenchymal stem cells (hBM-MSCs) showing spindle shaped morphology at 5th passage; (C) Growth kinetics of hfC-MSCs and hBM-MSCs seeded at a density of 1,000 cells per cm^2^.

We compared side by side the morphology of hfC-MSCs and hBM-MSCs under phase contrast microscopy and observed that similar to hBM-MSCs, hfC-MSCs grew as spindle shaped adherent cells **(Fig 1A and 1B).** We next evaluated the growth kinetics of hfC-MSCs and compared it to hBM-MSCs. We empirically chose 1000 cells/cm2 seeding density and counted the cell number of both the cell types, each day for 7 days. Interestingly, hfC-MSCs exhibited greater expansion potential as compared to BM-MSCs. Specifically, at day 7, the hfC-MSC cultures had more than 2-fold cell number compared to BM-MSCs, suggesting the superior proliferative ability of hfC-MSCs **(Fig 1C)**. Further, hfC-MSCs could be expanded to up to more than 80 population doublings with a population doubling time of approximately 34 hours. Human fC-MSCs displayed a normal karyotype over successive passages **(S1 Fig)**.

### Phenotypic characteristics and differentiation potential of hfC-MSCs

**Fig 2:**
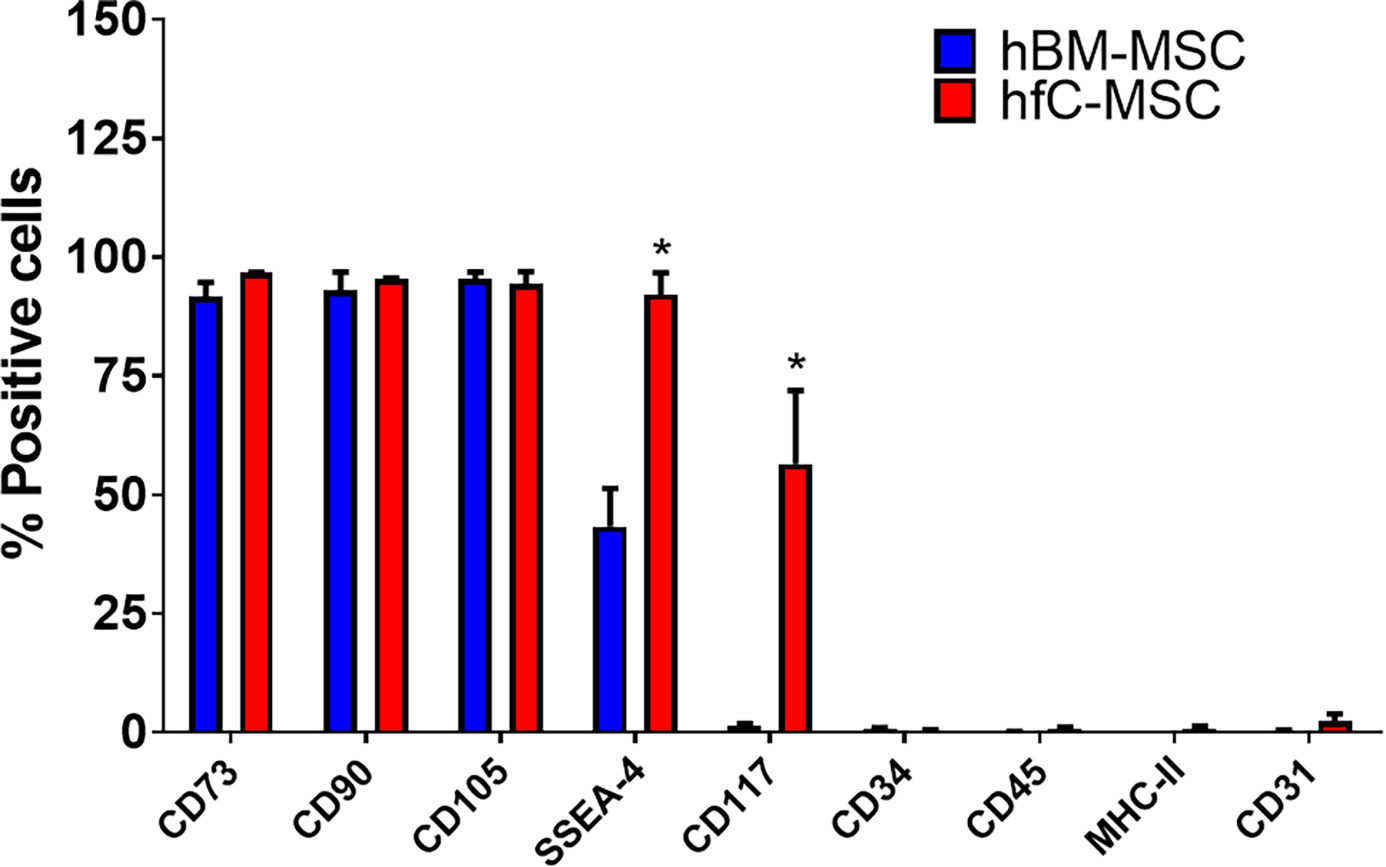
Phenotypic characteristics of hfC-MSCs compared to BM-MSCs. Representative bar graphs showing a comparison of the expression of CD73, CD90, CD105, SSEA-4, CD117, CD34, CD45, HLA-DR and CD31 on hfC-MSCs and hBM-MSCs as demonstrated by flow cytometry. Values are mean ± SE of three independent experiments of both the cell types at passage-5.

To evaluate the phenotype of the hfC-MSCs, we performed flow cytometric analysis. Our head to head comparison between hBM-MSCs and hfC-MSCs revealed that both the cell types expressed typical mesenchymal stem cell markers viz., CD73, CD90, CD105 whereas both cell types did not express CD31, CD34, CD45 and HLA-DR **(Fig 2).** However, fC-MSCs had significantly higher expression of SSEA-4 and c-kit compared to BM-MSCs, suggesting the primitive nature of the cells. Further, hfC-MSCs after treatment with adipogenic and osteogenic induction media could be induced to differentiate into adipocytes and osteoblasts as demonstrated by Oil red-O and Alizarin red staining, respectively **(S2 Fig)**.

### Expression of pluripotency and embryonic markers and cardiovascular genes by hfC-MSCs

**Fig 3:**
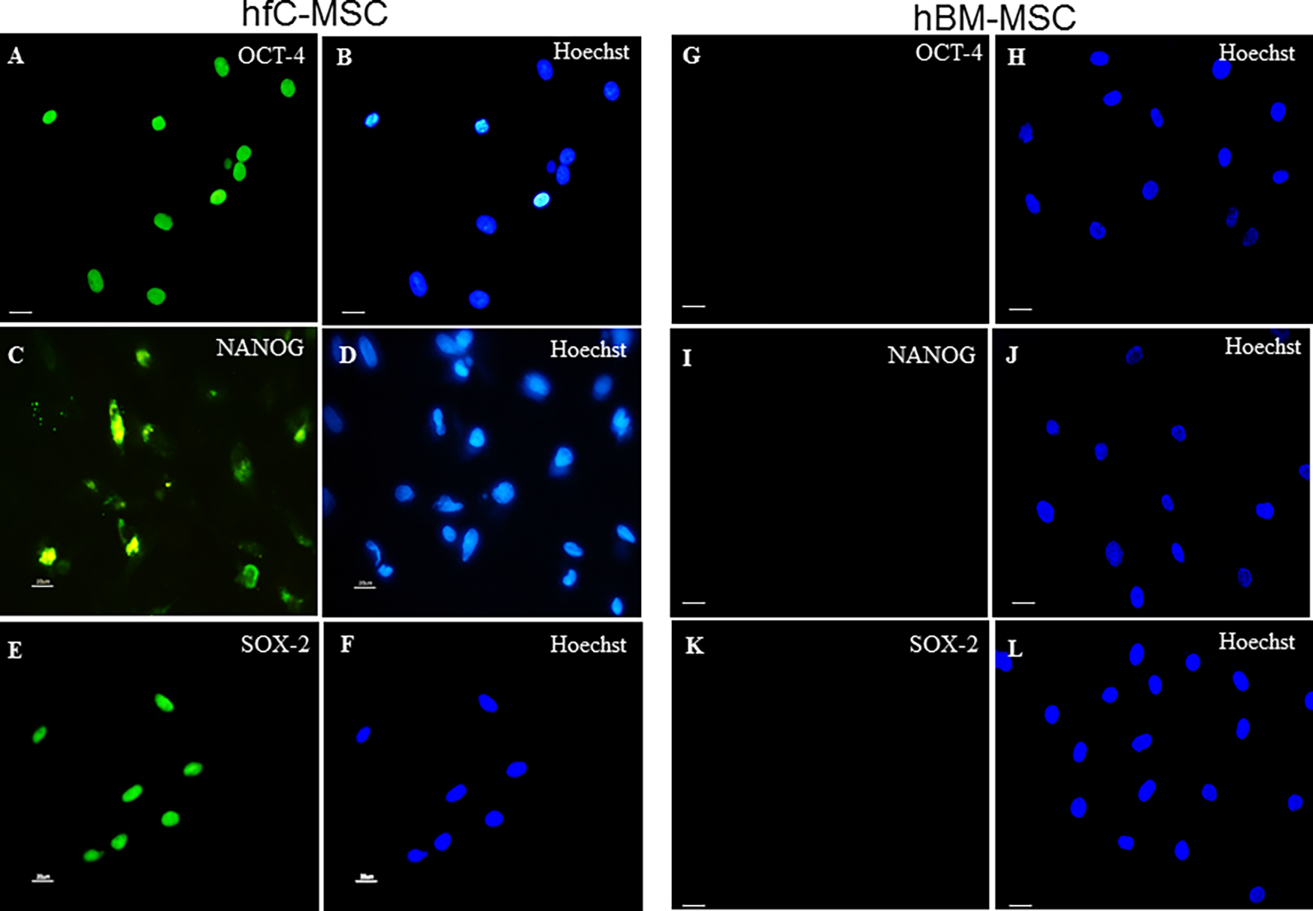
Expression of pluripotency and embryonic markers and cardiovascular genes by hfC-MSCs and hBM-MSCs. Representative photomicrographs (40X, 20μm) of human fetal cardiac mesenchymal stem cells (hfC-MSCs) showing expression of OCT-4 (A: OCT-4; B: hoechst dye), Nanog (C: Nanog; D: hoechst dye), SOX-2 (E: SOX-2; F: hoechst dye) and representative photomicrographs of (40X, 20μm) of human bone marrow mesenchymal stem cells (hfC-MSCs) showing expression of OCT-4 (G: OCT-4; H: hoechst dye), Nanog (I: Nanog; J: hoechst dye), SOX-2 (K: SOX-2; L: hoechst dye).

**Fig 4:**
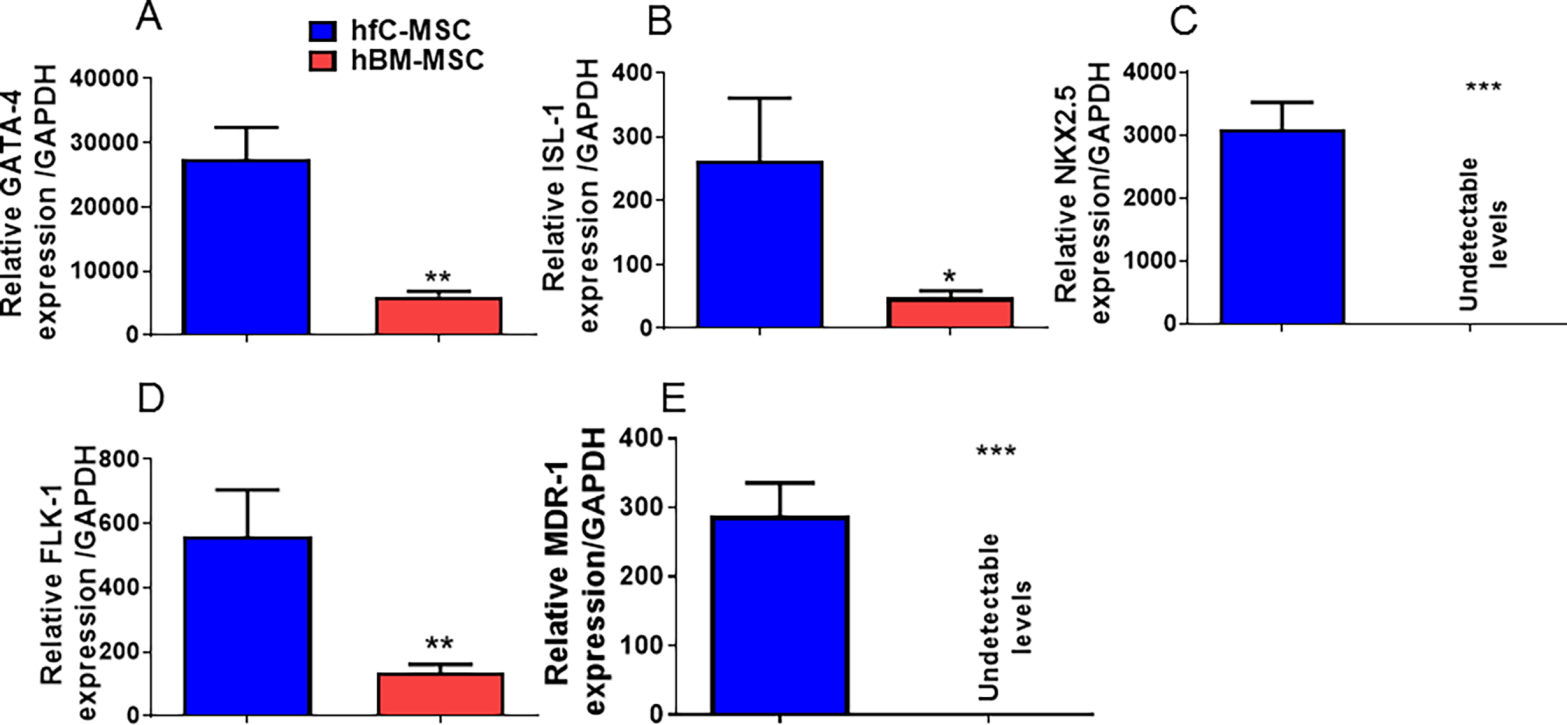
(A-E) Bar graphs showing relative gene expression of GATA-4, Isl-1, Flk-1, NKX2.5 and MDR-1 transcripts in human fetal cardiac mesenchymal stem cells (hfC-MSCs) compared to human bone marrow mesenchymal stem cells (hBM-MSCs) in 5^th^ passage (P5) as demonstrated by real time PCR. Data shown are mean ± SE of 3 experiments. *p<0.05 of hBM-MSCs vs human fC-MSCs.

We have previously shown that rat fC-MSCs express embryonal markers [12]. Similar to rat fC-MSCs, hfC-MSCs also expressed embryonal markers Oct-4, Nanog and Sox-2 as demonstrated by immunocytochemistry **(Fig 3A–3F).** However, the hBM-MSCs did not express these markers **(Fig 3G–3L),** highlighting the primitive nature of fC-MSCs. Furthermore hfC-MSCs consistently exhibited significantly elevated levels of GATA-4, flk-1, Isl-1 genes as compared to hBM-MSCs. Interestingly, NKX2.5 and MDR-1 expression levels were undetectable in the hBM-MSCs compared to hfC-MSCs **(Fig 4A–4E)**, suggesting the tissue specific gene expression in to hfC-MSCs.

### Differentiation of hfC-MSCs into cardiovascular cells

**Fig 5:**
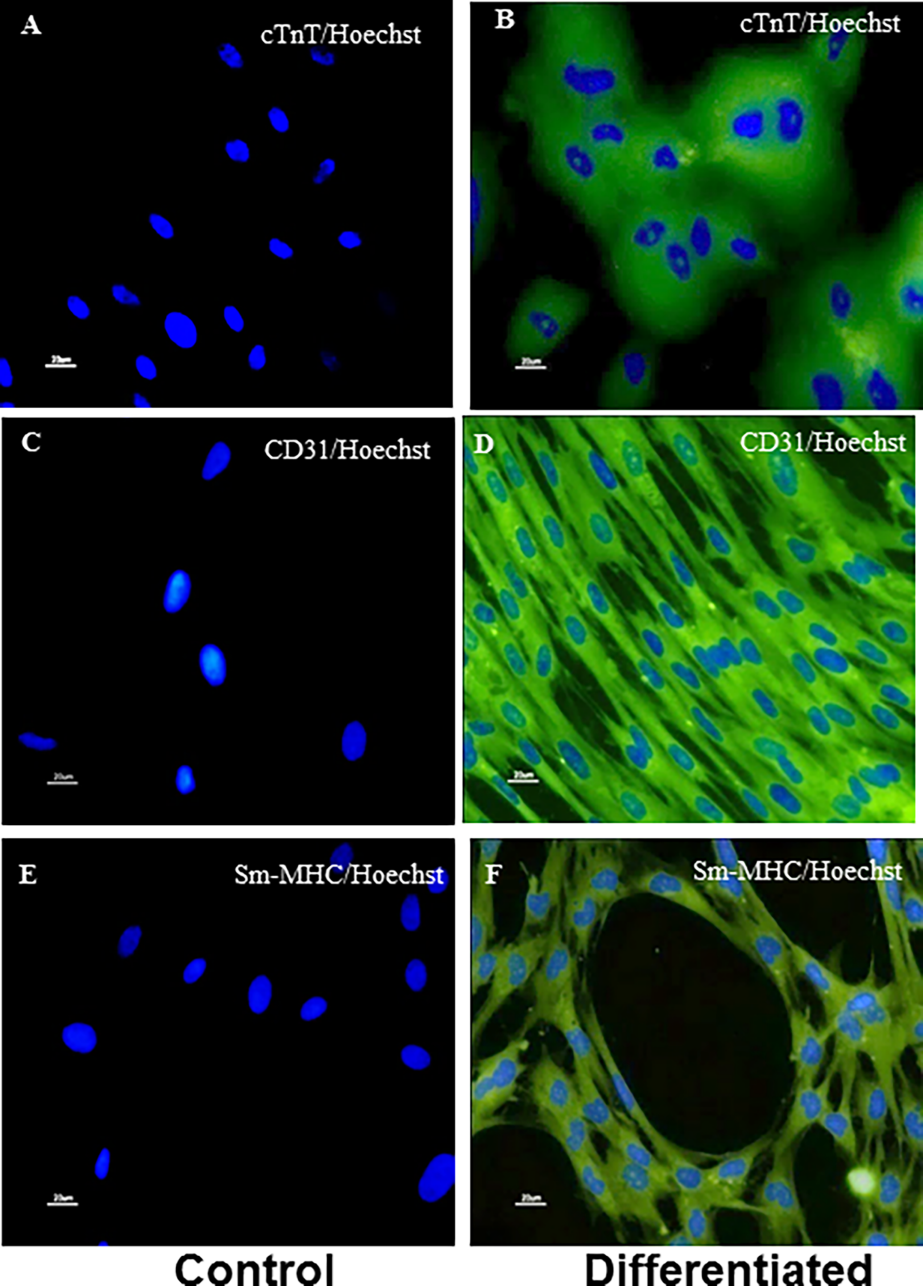
Differentiation of hfC-MSCs into cardiovascular cells. Representative immunocytochemistry images (40X, 20μm) showing differentiation of human fetal cardiac stem cells (hfC-MSCs) into Cardiomyocytes (B: Troponin-T (cTnT); Endothelial cells (D: CD31); Smooth Muscle Cells (F: Smooth Muscle- myosin heavy chain (SM-MHC); (A, C and E, were control cells without induction medium showing only hoechst dye). Data shown are from three independent experiments at passage 3–5.

Higher expression of cardiovascular markers by hfC-MSCs compared to hBM-MSCs prompted us to look at their cardiovascular differentiation ability. Upon induction, hfC-MSC could differentiate into major cardiovascular cell types’ viz. cardiomyocyte like cells, endothelial cells and smooth muscle cells (mesodermal lineage), as revealed by multinucleated differentiated cardiomyocytes with expression of cTnT (Fig 5A and 5B), CD31 on differentiated endothelial cells (Fig 5C and 5D) and SM-MHC on differentiated smooth muscle cells (**Fig 5E and 5F**).

### hfC-MSCs inhibit PHA-induced proliferation of lymphocytes

**Fig 6:**
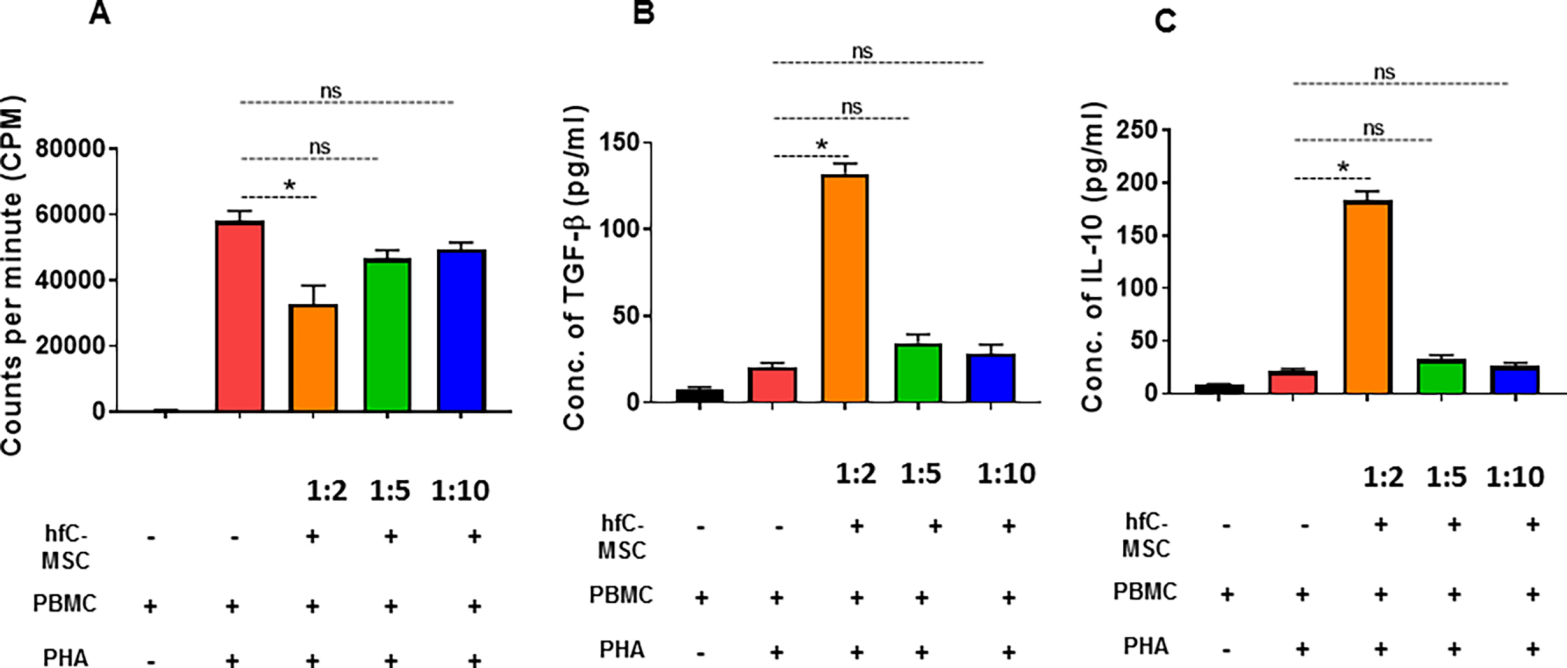
hfC-MSCs inhibit PHA-induced proliferation of lymphocytes. **(A)** PBMCs (1 × 10^5^ cells) stimulated with or without PHA (5 μg/ mL) in the presence or absence of irradiated hfC-MSCs (1 × 104–5 × 10^4^ cells). Data are expressed as the mean ± SE of three independent experiments. *p<0.05 (Control PBMCs vs. PBMCs+ MSCs), NS: Not significant. **(B)** TGF-β levels were analyzed in the culture supernatants of co-cultured hfC-MSCs and PBMCs stimulated with or without PHA. PBMCs (1 × 10^5^ cells) cultured with PHA (5 μg/mL) in the presence or absence of hfC-MSCs (1 × 104–5× 10^4^ cells). Data are expressed as the mean ± SE of three independent experiments. *p<0.05 (Control PBMC vs. PBMC+ MSC), NS: Not significant. **(C)** IL-10 levels were analysed in the culture supernatants of co-cultured hfC-MSCs and PBMCs stimulated with or without PHA. PBMCs (1 × 10^5^ cells) cultured with PHA (5 μg/mL) in the presence or absence of hfC-MSCs (1 × 104–5× 10^4^ cells). Data are expressed as mean ± SE of three independent experiments. *p<0.05 (Control PBMCs vs. PBMCs+ MSCs), NS: Not significant.

It has been recently shown that adult cardiac MSCs exhibit immunomodulatory properties [1]. Therefore, we assessed the immunomodulatory properties of hfC-MSCs. Our results revealed that hfC-MSCs inhibited PHA-induced dose dependent proliferation of PBMC. When hfC-MSCs were co-cultured with activated PBMCs at ratios of 1:2, PBMCs proliferation was significantly inhibited (*p* < 0.05) **(Fig 6A)**. However, when hfC-MSCs were co-cultured with PBMCs at a higher ratio of 1:5 and 1:10, there was no effect on PBMCs proliferation. Cytokines analysis revealed that hfC-MSCs secrete regulatory cytokines TGF-β and IL-10 to inhibit PBMCs proliferation (p<0.001) **(Fig 6B and 6C)**. These results strongly suggest that fC-MSCs exhibit immunomodulatory properties.

## Discussion

We have observed a novel population of mesenchymal stem cells in the heart of 14–16 weeks’ human fetus, that exhibited an extended self-renewal of up to more than 80 population doublings without any sign of senescence and karyotypic abnormality, expressed pluripotency markers namely Oct-4, Nanog, SOX-2 and SSEA-4 and exhibited an *in vitro* differentiation into cells of the three major cardiac lineages including cardiomyocyte like cells, endothelial cells and smooth muscle cells. The cells maintained a stable karyotype, undifferentiated state and multipotent differentiation potential over the successive passages. The hfC-MSCs markedly reduced T-lymphocyte proliferation with an increased secretion of TGF-β and IL-10. To the best of our knowledge this is the first report on the presence of multipotent mesenchymal stem cells exhibiting immunomodulation in human fetus heart.

We found that hfC-MSCs exhibited typical spindle shaped morphology and were adherent to plastic, similar to our published report on rfC-MSCs [12, 13] and one another independent observation in adult mouse C-MSCs [14]. Also similar to hBM-MSCs and rfC-MSCs, these cells expressed CD73, CD90, CD105, SSEA-4 but were negative for CD14, CD31, CD34, CD45 and MHC-II markers and could differentiate into adipocytes and osteocytes [12, 13]. Therefore, we labelled them as cardiac mesenchymal stem cells. However, SSEA-4 levels were significantly lower in the hBM-MSCs compared to hfC-MSCs, which might be due to the advancement of age. In agreement with previous reports [15, 16] hBM-MSCs in our hands also did not express C-kit. Further recent reports also suggest lack of c-kit expression on human fetal cardiovascular precursor cells and adult C-MSCs [14, 17]. However, our results reveal that hfC-MSCs express CD117 (C-Kit). This discrepancy of C-kit expression in different cardiac derived progenitor cells may be due to differences in the developmental stage or culture conditions or donor variability or loss of c-kit expression in vitro with increasing passages[18], needs to be further examined.

To further understand the differences between both hfC-MSCs and hBM-MSCs, we examined differences in the expression of cardiovascular genes including GATA-4, flk-1, Isl-1, NKX2.5 and MDR-1 by real time PCR. In corroboration with previous reports that different cardiac resident stem cells express specific cardiac markers [19], we found elevated levels of these cardiovascular genes in hfC-MSCs, suggesting the tissue specific expression and possible intrinsic propensity of fC-MSCs to differentiate into cardiovascular cells, compared to the BM-MSCs. Therefore, next we looked at the differentiation potential of hfC-MSCs into major cardiovascular cells including cardiomyocytes, endothelial cells and smooth muscle cells. Although we had previously shown the cardiovascular differentiation of MSCs from rat fetal heart [13], this study will be the first evidence that MSCs from human fetal heart could differentiate *in vitro* into cardiomyocyte like cells, endothelial cells and smooth muscle cells. A recent study has shown that, fetal cardiovascular precursor cells could only differentiate into cardiomyocytes and smooth muscle cells *in vitro* but not endothelial cells. This discrepancy between the two cells populations may be due to differences in the developmental stage of fetus hearts at which they had been obtained (12 vs 14–16 weeks) or variability within the pool of fetal cardiac stem cells. Further, future studies are warranted to investigate an optimal differentiation of hfC-MSCs into bona fide cardiomyocytes, which may also be utilized for drugs screening or cardio-toxicity studies.

We and others have previously shown that fetal mesenchymal stem cells from rodents and humans express embryonal markers [12, 20, 21]. Similarly, recent studies have also suggested that neonatal rat cardio sphere derived cells and adult human cardiac-MSCs express OCT-4, Nanog and Sox-2 [22, 23]. Moreover, heart is the first organ to be formed in embryogenesis and hfC-MSCs are likely to be most primitive MSCs with predilection for the expression of embryonal markers. In corroboration with above reports, our immunocytochemistry and flow cytometry studies revealed expression of embryonal markers including Oct-4, Nanog, SOX-2 and SSEA-4. Our previous study on rat fC-MSC transplantation did not result in teratogenicity in mouse models of MI. However, future studies are required to investigate teratogenicity of hfC-MSCs.

Mesenchymal stem cells are extensively studied cell types in regenerative medicine due to their immunomodulatory properties [24]. In line with previous reports suggesting the immunomodulatory properties of adult C-MSCs and fetal liver derived MSCs [1, 25], hfC-MSC also inhibited the proliferation of PBMCs via secretion of regulatory cytokines IL-10 and TGF-β. These results suggest that hfC-MSCs could be a potential off the shelf allogenic source for cardiovascular regenerative medicine.

## Conclusion

In conclusion, this study has demonstrated that human fetus heart has a population of primitive MSCs that express embryonal markers and are committed towards cardiovascular lineage, making them an attractive cell type for cardiovascular regenerative therapy. Further, these cells exhibited intense immunomodulatory properties, suggesting that they could be a potential off the shelf allogenic source of cells for therapy. We do acknowledge that aborting of fetuses may not be legally permitted in many countries and therefore the accessibility to these tissue sources may be questioned. However, our finding could be useful in countries where abortion or medical termination of pregnancy is legal. Further studies on the therapeutic efficacy in pre-clinical animal models of cardiac diseases, will be important to bring out the translational value of these stem cells.

## Supporting information

S1 Fig Normal karyotype of fC-MSCs at 15^th^ passage. Representative photomicrographs showing normal karyotype of human fetal cardiac mesenchymal stem cells at 15th passage.
(TIF)

S2 Fig fC-MSCs differentiation into adipocytes and osteoblasts. Representative photomicrographs (10X, 20μm) showing differentiation of human fetal cardiac mesenchymal stem cells into adipocytes and osteocytes: (A) control cells showing no stain; (B) cells differentiated into adipocytes positive for Oil red O; (C) control cells showing no stain; D) cells differentiated into osteocytes positive for Alizarin red stain.
(TIF)

## Abbreviations

MSC: Mesenchymal stem cells
fC-MSCs: Fetal cardiac mesenchymal stem cells
BM-MSCs: Bone marrow mesenchymal stem cells
C-MSCs: Cardiac mesenchymal stem cells
LV: left ventricle
SSEA-4: Stage specific embryonic antigen-4
ISL-1: Insulin gene enhancer protein
MDR-1: Multi drug resistance gene-1
FITC: Fluorescence isothyocyanate
PE: Phycoerythrin
PBMC: peripheral blood mononuclear cells
TGF-β: Transforming growth factor receptor-β
IL-10: Interlukin-10
PHA: Phytohemagglutinin
