# Supplementary figures and images for: Isolation and characterization of mesenchymal stem cells from human fetus heart
Source: PLoS One. 2018 Feb 8;13(2):e0192244. doi: 10.1371/journal.pone.0192244 (PMC5805293; doi:10.1371/journal.pone.0192244)

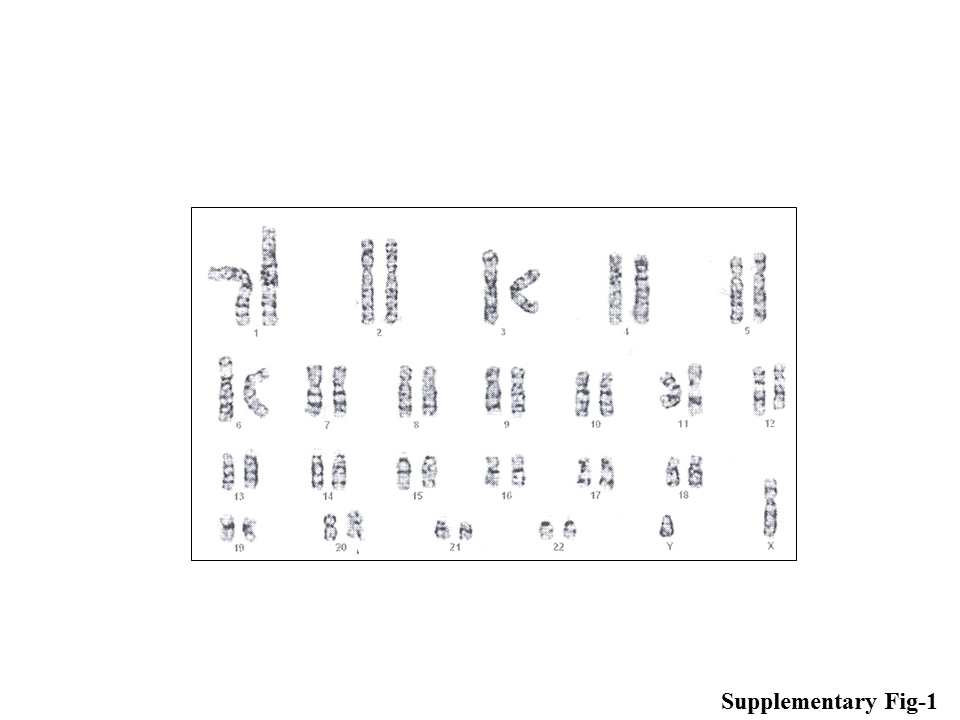

Supplement: S1 Fig — Representative photomicrographs showing normal karyotype of human fetal cardiac mesenchymal stem cells at 15th passage. (TIF) [file pone.0192244.s001.tif]

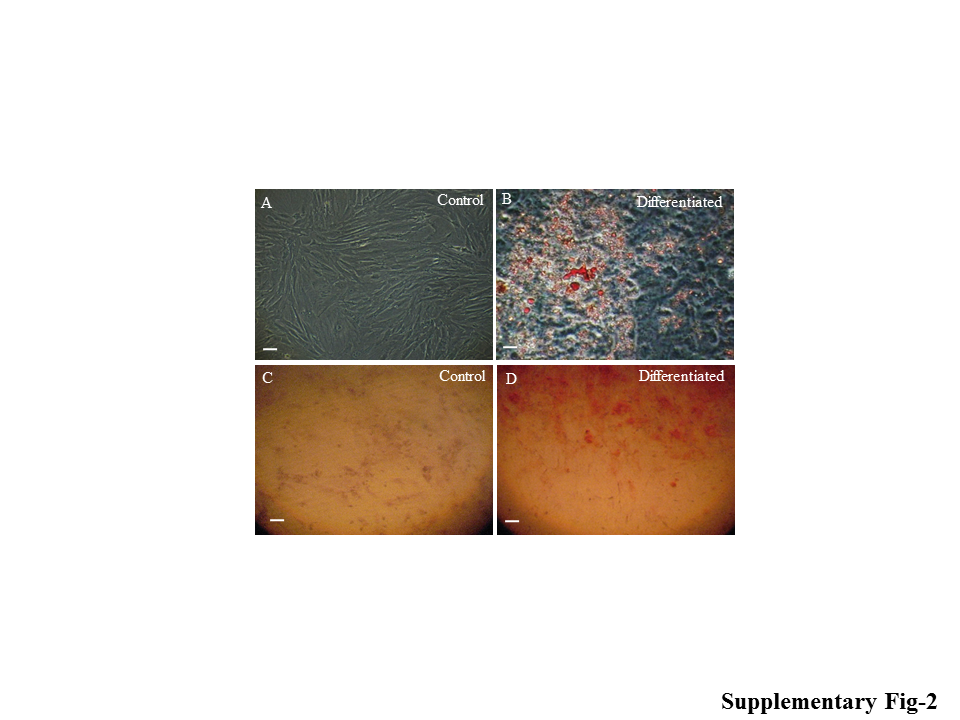

Supplement: S2 Fig — Representative photomicrographs (10X, 20μm) showing differentiation of human fetal cardiac mesenchymal stem cells into adipocytes and osteocytes: (A) control cells showing no stain; (B) cells differentiated into adipocytes positive for Oil red O; (C) control cells showing no stain; D) cells differentiated into osteocytes positive for Alizarin red stain. (TIF) [file pone.0192244.s002.tif]
